# Dietary intakes of branched-chain amino acids and plasma lipid profiles among filipino women in Korea: the Filipino Women’s Diet and Health Study (FiLWHEL)

**DOI:** 10.1186/s12937-023-00861-w

**Published:** 2023-07-11

**Authors:** Akinkunmi Paul Okekunle, Heejin Lee, Sherlyn Mae P. Provido, Grace H. Chung, Sangmo Hong, Sung Hoon Yu, Chang Beom Lee, Jung Eun Lee

**Affiliations:** 1 Department of Food and Nutrition, College of Human Ecology, Seoul National University, 1 Gwanak- ro, Gwanak-gu, Seoul, 08826 Korea; 2 Research Institute of Human Ecology, Seoul National University, 1 Gwanak-ro, Gwanak-gu, Seoul, 08826 Korea; 3 Department of Child Development & Family Studies, Seoul National University, 1 Gwanak-ro, Gwanak-gu, Seoul, 08826 Korea; 4 Division of Endocrinology and Metabolism, Department of Internal Medicine, Hanyang University Guri Hospital, Hanyang University College of Medicine, 153 Gyeongchun-ro, Guri, 11923 Korea

**Keywords:** Diets, Branched-chain amino acids, Total cholesterol, Triglycerides, High-density lipoprotein cholesterol, Low-density lipoprotein cholesterol, FiLWHEL

## Abstract

**Background:**

The potential role of dietary branched-chain amino acids (BCAA) in metabolic health, including cardiovascular disease and diabetes, is evolving, and it is yet to be understood if dietary BCAA intakes are associated with plasma lipid profiles or dyslipidaemia. This study tested the association of dietary BCAA intakes with plasma lipid profiles and dyslipidaemia among Filipino women in Korea.

**Methods:**

Energy-adjusted dietary BCAA intakes (isoleucine, leucine, valine, and total BCAA) and fasting blood profiles of triglycerides (TG), total cholesterol (TC), high-density lipoprotein-cholesterol (HDL-C), and low-density lipoprotein-cholesterol (LDL-C) were determined in a sample of 423 women enrolled in the Filipino Women’s Diet and Health Study (FiLWHEL). The generalized linear model was applied to estimate least-square (LS) means and 95% confidence intervals (CIs) and compare plasma TG, TC, HDL-C, and LDL-C across tertile distribution of energy-adjusted dietary BCAA intakes at *P* < 0.05.

**Results:**

Mean of energy-adjusted dietary *total* BCAA intake was 8.3 ± 3.9 g/d. Average plasma lipid profiles were 88.5 ± 47.4 mg/dl for TG, 179.7 ± 34.5 mg/dl for TC, 58.0 ± 13.7 mg/dl for HDL-C, and 104.0 ± 30.5 mg/dl for LDL-C. LS means, and 95% CIs across tertiles of energy-adjusted *total* BCAA intakes were 89.9 mg/dl, 88.8 mg/dl and 85.8 mg/dl (*P*-trend = 0.45) for TG, 179.1 mg/dl, 183.6 mg/dl and 176.5 mg/dl (*P*-trend = 0.48) for TC, 57.5 mg/dl, 59.6 mg/dl and 57.1 mg/dl (*P*-trend = 0.75) for HDL-C and 103.6 mg/dl, 106.2 mg/dl and 102.3 mg/dl (*P*-trend = 0.68) for LDL-C. Furthermore, the multivariable-adjusted prevalence ratios and 95% confidence intervals for dyslipidaemia across increasing tertile distribution of energy-adjusted *total* BCAA intake were; 1.00, 0.67 (0.40, 1.13) and 0.45 (0.16, 1.27; *P-trend* = 0.03) for the first, second and third tertile, respectively.

**Conclusions:**

Higher dietary intakes of BCAA presented a statistically significant inverse trend with the prevalence of dyslipidaemia among Filipino women in this study and testing these associations in longitudinal studies may be necessary to confirm these findings.

**Supplementary Information:**

The online version contains supplementary material available at 10.1186/s12937-023-00861-w.

## Introduction

The role of branched-chain amino acids (BCAAs), isoleucine, leucine, and valine, cannot be underscored in metabolic processes and nutrient signalling [1–4]. They are primarily from food sources of proteins such as eggs, fish, meats, and milk [3]. They account for more than fifty percent of all essential amino acids food supply in mammals [5], and higher consumption of BCAAs has been linked to higher odds of diabetes mellitus [6–9], with diet quality playing a critical role in that relationship [8–10]. For example, dietary BCAA was associated with higher odds of diabetes mellitus among those reporting higher meat consumption [9] but not among those who reported higher consumption of plant-based diets [10].

Disturbances in glucose metabolism can negatively affect lipid and fatty acid metabolism [11, 12], but the significance of BCAA in this complex glucose-lipid metabolism is yet to be unravelled. It is yet to be clearly understood whether higher dietary BCAA intake can manipulate lipid metabolism. Some studies have reported that elevated circulating BCAA concentrations confers a significant cumulative risk of developing metabolic disorders [13], including dyslipidaemia [14–16], but if dietary BCAA is associated with dyslipidaemia remains unclear. For example, a previous study demonstrated that plasma BCAAs were positively correlated with higher odds of disordered lipid profiles in a Chinese population [15] without clarifying the role of dietary BCAAs in the observed associations.

Dyslipidaemia is a constellation of disorders attributable to the accretion of unfavourable lipids [11, 17] with adverse health consequences and a significant burden on the cost of care [18]. It is a multifaceted metabolic disorder that predominantly interfaces with environmental, lifestyle, and genetic factors [19]. While several epidemiological studies have reported the association of dietary and circulating BCAA with chronic diseases such as diabetes [6, 13] and obesity [13, 20, 21], there is limited data on the association of dietary BCAA with plasma lipid profiles and dyslipidaemia. Whether dietary intakes of BCAAs can interact with the complex architecture of lipid metabolism in the vasculature to promote the accumulation of unfavourable lipids is yet to be well understood.

Discerning the relationship of dietary BCAA with dyslipidaemia is vital for extending the frontiers of understanding on the role of dietary and circulation BCAAs in metabolic disorders. Such information is likely to guide dietary recommendations and public health interventions for the primary prevention and management of metabolic disorders. In a broader context, nutrients (such as the dietary BCAAs) are consumed in the complex conundrum of dietary exposures associated with metabolic disorders. It would be worthwhile to account for the role of diet quality in assessing the relationship of dietary BCAAs with plasma lipid profiles and dyslipidaemia.

This study hypothesizes that dietary BCAAs, including isoleucine, leucine and valine, are associated with dyslipidaemia. Similarly, we hypothesize that dietary BCAAs are associated with plasma lipid profiles; triglycerides (TG), total cholesterol (TC), high-density lipoprotein cholesterol (HDL-C) and low-density lipoprotein cholesterol (LDL-C). Therefore, this study tested the association of dietary BCAA intakes with plasma lipid profiles and dyslipidaemia among Filipino women in Korea.

## Methods

### Study population

We analyzed the relationship between dietary BCAAs and plasma lipid profiles among women in the Filipino women’s diet and health (FiLWHEL) study in Korea. The FiLWHEL study is an ongoing study among Filipino women in Korea. At baseline, it commenced in 2014 to characterize the contribution(s) of health-related behaviour(s), lifestyle and acculturation among Filipino women in Korea. The Institutional Review Board of Sookmyung Women’s University (SMWU-1311-BR-012) approved the study, and all respondents gave written informed consent. Convenience sampling was adopted to recruit respondents from some cities in Korea. Complete information on the protocol, recruitment [22] and primary findings [23–25] in the FiLWHEL study has been reported elsewhere [22–25].

Information on demographic characteristics, health-related behaviour, medical history, quality of life, and acculturation were provided by respondents using interviewer-administered questionnaires. Respondents’ height and waist circumference (WC) was measured to the nearest 0.1 cm using a stretch-resistant tape rule, and weight (in kg) was measured using bioelectric impedance equipment (In Body 620, Biospace Company Limited, Seoul, Korea). Body mass index (BMI) was calculated as weight (kg) divided by the square of height (m), and obesity was defined as BMI ≥ 25 kg/m^2^ according to the recommendations of the World Health Organization (Western Pacific Regional Office), the International Association for the Study of Obesity, and the International Obesity task force [26].

Furthermore, respondents recalled diet information using a 24-hour recall, and portion sizes were projected using food miniatures, photographs, household measures, weight/volume, and standard units and portions. Trained Filipino volunteers conducted all interviews with the same protocol in all sites under the supervision of the principal investigators. All information on the questionnaire was inspected on-site. Before data coding, questionnaires were rechecked, inconsistencies were clarified over the phone, and codes were double-checked for data reliability. A trained phlebotomist drew fasting blood samples from respondents after at least 8h of overnight fast. Blood samples were centrifuged and kept at 2-8^0^ C before processing, and storage (at -86^0^C). Out of the 504 women enumerated in the study, 81 respondents were excluded; pregnant and lactating (n = 68) or those with missing information (24-hour recall, n = 07, and anthropometry, n = 06). Finally, 423 respondents were included in the final analysis of this study.

### Determination of lipid profiles and definition of dyslipidaemia (Outcome)

TG, TC and HDL-C in milligrams per decilitre were determined by Seegene Medical Foundation (Seoul, Korea) using the Cobas 8000 C702-I (Roche Diagnostics, Basel, Switzerland) [27]. Also, using the Friedewald Eq.  [28], we estimated the LDL-C profiles. Coefficients of variations of samples for the lipid profile analysis were typically below 3%. D*yslipidemia* was defined as one of the following conditions; a previous diagnosis of elevated lipid profiles, current use of statins or lipid-lowering medications, TG ≥ 150 mg/dl, TC ≥ 200 mg/dl, HDL-C < 50 mg/dl or LDL-C ≥ 130 mg/dl according to *the* National Cholesterol Education Program Adult Treatment Panel III NCEP-ATP (III) guidelines [29].

### Dietary BCAA intake assessment (Exposure)

Dietary BCAAs; isoleucine, leucine, and valine were estimated from food and dietary information using 24-hour recalls, and total BCAA (*t*BCAA) was computed as a sum of isoleucine, leucine, and valine. Respondents provided information on all food items, portion sizes and food amount consumed on the previous day preceding the survey. Nutrient data were computed and transformed into grams per day (g/d) using the computer-aided analysis program 4.0 for professionals by the Korean Society of Nutrition, Seoul, Korea [30]. Where food information was unavailable, the food composition tables of the Food and Nutrition Research Institute of the Philippines (for Filipino diets) [31], Korean Rural Development Administration [32], United States Department of Agriculture [33] or manufacturers’ information was used to derive nutrient information.

### Estimation of diet quality scores

Diet quality was estimated using the minimum dietary diversity for women (MDD-W) developed by the Food and Agriculture Organization. The MDD-W was developed as a proxy for micronutrient adequacy among women of reproductive age from low and middle-income countries in the women’s dietary diversity project [34]. Ten [10] food groups, including grains, white roots, and tubers; pulses (beans, peas, and lentils); nuts and seeds; dairy; meat, poultry, and fish; eggs; dark green leafy vegetables; other vitamin A-rich fruits and vegetables; other vegetables; and other fruits were used in computing the MDD-W score. Each respondent was allotted a unit point score for a minimum dietary intake of 15 g (one serving) or more for each food group; otherwise, zero points and the summation of the scores (which ranged from 0 to 10) represented the overall MDD-W. The higher the MDD-W score, the better the dietary quality of respondents. Details of how the MDD-W scores were estimated have been reported elsewhere [34, 35].

### Demographic and lifestyle characteristics (Covariates)

Respondents offered information on age (in years and dichotomized as < 35 and ≥ 35 years using the median age), length of stay in Korea [in years and categorized ≤ 4 years (25th percentile), 5–9 years (> 25th – 75th) and 10 years (≥ 75th percentile)], level of education (‘Elementary and high school’ and ‘College education and above’), employment status (‘no’ and ‘yes’), ever smoked 100 cigarettes in a lifetime (‘no’ and ‘yes’) and current alcohol use (‘no’ and ‘yes’). The average number of hours and the number of days spent on physical activity (moderate, vigorous or walking) were provided and vigorous physical activity was defined as having spent at least an hour daily of vigorous physical activity [36]. Respondents were asked if they were diagnosed with diabetes or hypertension by a certified clinician or are currently taking medications to lower blood glucose or blood pressure.

### Statistical analysis

Isoleucine, leucine, valine, and *t*bCAA were adjusted for energy intake using the residual method [37, 38] and categorized into tertiles to include a reasonable number of respondents in each category. Characteristics of respondents were presented across tertile distribution of energy-adjusted dietary BCAA intakes. Multivariable-adjusted regression was applied to estimate the prevalence ratios (PRs) and 95% confidence intervals (CIs) of having dyslipidaemia by tertile distribution of energy-adjusted dietary BCAA intakes. We assessed changes in PRs when deciding on variables to be included in the final model. In model 1, we adjusted for age (continuous years), years of stay in Korea (≤ 4 years, 5–9 years, ≥ 10 years), education (elementary and high school, college education and above), employment status (no, yes), ever smoked 100 cigarettes in a lifetime (no, yes), current alcohol use (no, yes) and energy intakes (continuous, kcal/d). Model 2 was adjusted for vigorous physical activity (no, yes) in addition to variables in model 1. Model 3 was adjusted for a history of diabetes (no, yes) and a history of hypertension (no, yes) in addition to variables in model 2. In model 4, we adjusted for BMI (continuous, kg/m^2^) in addition to variables in model 3. In model 5, we adjusted for MDD-W scores (continuous) in addition to covariates in model 4. Test for trend was carried out by assigning the median value of tertile distribution as a continuous variable in the model. Furthermore, we estimated least-square (LS) means and 95% confidence interval (CIs) of total TG, TC, HDL-C and LDL-C across tertile distribution of energy-adjusted dietary BCAA intakes using a generalized linear model adjusting for the same covariates. All food items were classified into 21 food groups (fruits, vegetables, tubers, nuts and seeds, grains, noodles, pasta, bread, snacks, sugars, meat, fast foods, fish, seafood, eggs, poultry, beverages, dairy, condiment, oils, and alcohol; based on macronutrient composition and mode of preparation), and percentage contribution of each food group to dietary isoleucine, leucine and valine intakes was estimated by weighting the total of each BCAA on the overall sum of the BCAA in all food groups. Details of the estimation method have been reported elsewhere [39, 40]. All statistical analyses were conducted using SAS 9.4 (SAS Institute Inc., Cary, NC, USA) at *P* < 0.05.

## Results

Overall, mean ± standard deviation(SD) of energy-adjusted dietary BCAA intake was 8.3 ± 4.9 g/d for *t*BCAA, 2.1 ± 1.3 g/d for isoleucine, 3.7 ± 2.2 g/d for leucine, and 2.5 ± 1.5 g/d for valine. Also, the mean ± SD of lipid profiles was 88.5 ± 47.4 mg/dl for total TG, 179.7 ± 34.5 mg/dl for TC, 58.0 ± 13.7 mg/dl for HDL-C, 104.0 ± 30.5 mg/dl for LDL-C and 208 (49.2%) had dyslipidaemia.

Characteristics of respondents by tertile distribution of energy-adjusted dietary *t*BCAA intake are presented in Table 1. The proportion of those ≥ 35 years in the first, second, and third tertile of energy-adjusted dietary tBCAA intake was 67 (47.5%), 72 (51.1%), and 76 (53.9%), respectively. The proportion of those employed increased across tertiles of energy-adjusted dietary *t*BCAA intake; 71 (50.3%), 76 (53.6%) and 84 (59.3%) for the first, second and third tertile, respectively. Mean BMI and WC differed insignificantly across tertile distribution of energy-adjusted dietary tBCAA intakes. The mean of energy-adjusted *t*BCAA intakes by tertile distribution of energy-adjusted *t*BCAA intakes were 4.4 ± 1.9* g/d*, 8.0 ± 0.8* g/d* and 12.5 ± 3.1* g/d* for first, second and third tertile, respectively. Also, mean intakes of energy-adjusted individual dietary BCAA, by tertile distribution of energy-adjusted dietary *t*BCAA, were 1.1 ± 0.5* g/d*, 2.1 ± 0.2* g/d* and 3.2 ± 0.8* g/d* for isoleucine, 1.9 ± 0.8* g/d*, 3.6 ± 0.4* g/d* and 5.6 ± 1.5* g/d* for leucine and 1.3 ± 0.6* g/d*, 2.4 ± 0.3* g/d* and 3.7 ± 0.9* g/d* for valine across the first, second and third tertile, respectively. Similarly, mean MDD-W scores were 6.0 ± 1.9, 6.5 ± 1.7 and 6.8 ± 1.5 for the first, second and third tertile of energy-adjusted *t*BCAA intake, respectively. Mean energy intakes were; 1844.4 ± 799.1 *kcal/d*, 1558.1 ± 588.0 *kcal/d* and 1810.1 ± 605.8 *kcal/d* for the first, second and third tertile of energy-adjusted dietary tBCAA intake, respectively. Prevalences of dyslipidaemia by tertile distribution of energy-adjusted *t*BCAA intake were 76 (53.9%), 67 (47.5%) and 65 (46.1%) for the first, second and third tertile, respectively. Characteristics of respondents by tertile distribution of energy-adjusted dietary isoleucine (Table S1), leucine (Table S2) and valine (Table S3) intakes followed a similar trend as the tertile distribution of energy-adjusted dietary *t*BCAA intake. Primary food sources of dietary BCAA in this population are presented in Table S4. Dietary contributions to BCAAs were highest for red meat (isoleucine – 23.8%, leucine – 24.8%, valine – 24.8%), grains (isoleucine – 19.2%, leucine – 20.8%, valine – 17.9%) and fish (isoleucine – 11.7%, leucine – 10.8%, valine – 11.1%).

**Table 1:** Characteristics of Filipino women by tertile distribution of energy-adjusted total BCAA intake in the FiLWHEL study

	Tertile distribution of energy-adjusted *total* BCAA intake		
Characteristics	Tertile 1	Tertile 2	Tertile 3
*N*	141	141	141
Age (years)	34.4 ± 8.1	35.7 ± 8.3	35.6 ± 7.4
< 35 years	74 (52.5)	69 (48.9)	65 (46.1)
≥ 35 years	67 (47.5)	72 (51.1)	76 (53.9)
Length of stay (years)	7.8 ± 5.1	7.5 ± 4.6	7.7 ± 4.9
≤ 4 years	44 (31.2)	41 (29.1)	39 (27.6)
5–9 years	49 (33.7)	59 (41.8)	62 (44.1)
≥ 10 years	48 (34.1)	41 (29.1)	40 (28.3)
Education			
Elementary and high school	44 (31.4)	42 (30.0)	44 (31.4)
College education and above	97 (68.6)	99 (70.0)	97 (68.6)
Employment status (*Yes*)	71 (50.3)	76 (53.6)	84 (59.6)
Ever smoked (*Yes*)	12 (8.6)	9 (6.5)	15 (10.8)
Current alcohol use (*Yes*)	84 (59.5)	83 (58.9)	80 (56.7)
Vigorous physical activity (*Yes*)	31 (22.1)	22 (15.6)	26 (18.4)
History of diabetes* (*Yes*)	2 (1.4)	2 (1.4)	6 (4.3)
History of hypertension* (*Yes*)	11 (7.9)	9 (6.4)	9 (6.4)
BMI (kg/m^2^)	23.7 ± 3.8	23.4 ± 3.6	23.7 ± 4.3
≥ 25 kg/m^2^	48 (34.0)	42 (29.8)	38 (27.3)
Waist circumference (cm)	79.4 ± 8.8	79.0 ± 8.8	79.7 ± 10.4
≥ 80 cm	60 (42.6)	63 (44.7)	55 (39.0)
Total energy (*kcal/d*)	1844.4 ± 799.1	1558.1 ± 588.0	1810.1 ± 605.8
Dietary total BCAA intake (*g/d*)^†^	4.4 ± 1.9	8.0 ± 0.8	12.5 ± 3.1
Isoleucine intake (*g/d*)^†^	1.1 ± 0.5	2.1 ± 0.2	3.2 ± 0.8
Leucine intake (*g/d*)^†^	1.9 ± 0.8	3.6 ± 0.4	5.6 ± 1.5
Valine intake (*g/d*)^†^	1.3 ± 0.6	2.4 ± 0.3	3.7 ± 0.9
MDD-W score	6.0 ± 1.9	6.5 ± 1.7	6.8 ± 1.5
TG (mg/dl)	87.3 ± 44.1	90.2 ± 46.6	87.9 ± 51.6
≥ 150 mg/dl	13 (9.3)	14 (10.1)	14 (10.5)
TC (mg/dl)	177.6 ± 33.9	184.3 ± 34.2	177.1 ± 35.2
≥ 200 mg/dl	35 (25.0)	44 (31.9)	32 (23.9)
HDL-C (mg/dl)	57.7 ± 13.9	59.4 ± 13.8	57.0 ± 13.4
≤ 50 mg/dl	42 (30.0)	30 (21.7)	41 (30.6)
LDL-C (mg/dl)	102.4 ± 31.4	106.9 ± 29.7	102.5 ± 30.2
≥ 130 mg/dl	23 (16.4)	30 (21.7)	23 (17.2)
Dyslipidaemia^‡^ (*Yes*)	76 (53.9)	67 (47.5)	65 (46.1)

mean ± SD for continuous variables and n (%) for categorical variables

*Self-reported clinical diagnosis or current use of medication; BMI: body mass index; MDD-W: minimum dietary diversity for women; TG: triglycerides; TC: total cholesterol; HDL-C: high-density lipoprotein cholesterol; LDL-C: low-density lipoprotein cholesterol

^†^Amino acid intake was adjusted for energy intake using the residual method

^‡^D*yslipidemia* was defined as one of the following conditions; a previous diagnosis of elevated lipid profiles, current use of statins or lipid-lowering medications, TG ≥ 150 mg/dl, TC ≥ 200 mg/dl, HDL-C < 50 mg/dl or LDL-C ≥ 130 mg/dl according to *the* NCEP-ATP (III) guidelines.

Multivariable-adjusted PR and 95%CIs of the associations between energy-adjusted dietary BCAA intakes and dyslipidaemia are presented in Table 2. Multivariable-adjusted PRs and 95% CIs for dyslipidaemia across tertile distribution of energy-adjusted *t*BCAA intake were; 1.00, 0.67 (0.40, 1.13) and 0.45 (0.16, 1.27; *P trend* = 0.03) for the first, second and third tertile, respectively after adjusting for age, years of stay in Korea, education, employment, ever smoked, current alcohol use, total energy intake, vigorous physical activity, history of diabetes or hypertension, BMI and MDD-W scores. A similar trend was observed by tertile distribution of energy-adjusted dietary isoleucine [first tertile – 1.00, second tertile – 0.77 (0.46, 1.29) and third tertile – 0.59 (0.21, 1.65), *P trend* = 0.07], leucine [first tertile – 1.00, second tertile – 0.75 (0.45, 1.27) and third tertile – 0.57 (0.20, 1.62), *P trend* = 0.04] and valine [first tertile – 1.00, second tertile – 0.66 (0.39, 1.11) and third tertile – 0.43 (0.15, 1.22), *P trend* = 0.02] intakes.

**Table 2:** Prevalence ratios and 95% confidence intervals for the dyslipidaemia* by tertile distribution of energy-adjusted dietary BCAA intakes†

	Tertile 1	Tertile 2	Tertile 3	*P for trend*
*total* BCAA				
cases/total	76/141	67/141	65/141	
Age-adjusted	1.00	0.75 (0.47, 1.20)	0.57 (0.22, 1.45)	0.16
Model 1	1.00	0.73 (0.45, 1.20)	0.50 (0.20, 1.43)	0.11
Model 2	1.00	0.73 (0.45, 1.19)	0.53 (0.20, 1.41)	0.11
Model 3	1.00	0.73 (0.45, 1.19)	0.53 (0.20, 1.42)	0.09
Model 4	1.00	0.75 (0.45, 1.25)	0.57 (0.21, 1.56)	0.08
Model 5	1.00	0.67 (0.40, 1.13)	0.45 (0.16, 1.27)	0.03
Isoleucine				
cases/total	74/141	69/141	65/141	
Age-adjusted	1.00	0.85 (0.53, 1.36)	0.72 (0.28, 1.85)	0.25
Model 1	1.00	0.85 (0.52, 1.40)	0.73 (0.27, 1.94)	0.17
Model 2	1.00	0.85 (0.52, 1.39)	0.72 (0.27, 1.93)	0.17
Model 3	1.00	0.85 (0.52, 1.39)	0.72 (0.27, 1.92)	0.15
Model 4	1.00	0.85 (0.51, 1.41)	0.72 (0.26, 1.99)	0.16
Model 5	1.00	0.77 (0.46, 1.29)	0.59 (0.21, 1.65)	0.07
Leucine				
cases/total	75/141	69/141	64/141	
Age-adjusted	1.00	0.82 (0.51, 1.32)	0.68 (0.26, 1.73)	0.16
Model 1	1.00	0.79 (0.48, 1.30)	0.63 (0.24, 1.70)	0.13
Model 2	1.00	0.79 (0.48, 1.30)	0.62 (0.23, 1.68)	0.13
Model 3	1.00	0.79 (0.48, 1.30)	0.63 (0.23, 1.69)	0.11
Model 4	1.00	0.84 (0.50, 1.40)	0.70 (0.25, 1.96)	0.10
Model 5	1.00	0.75 (0.45, 1.27)	0.57 (0.20, 1.62)	0.04
Valine				
cases/total	76/141	67/141	65/141	
Age-adjusted	1.00	0.75 (0.47, 1.19)	0.55 (0.22, 1.43)	0.16
Model 1	1.00	0.75 (0.46, 1.22)	0.56 (0.21, 1.49)	0.12
Model 2	1.00	0.74 (0.45, 1.21)	0.55 (0.20, 1.47)	0.11
Model 3	1.00	0.74 (0.45, 1.22)	0.55 (0.21, 1.48)	0.10
Model 4	1.00	0.73 (0.44, 1.21)	0.53 (0.19, 1.47)	0.08
Model 5	1.00	0.66 (0.39, 1.11)	0.43 (0.15, 1.22)	0.02

^*^D*yslipidemia* was defined as one of the following conditions; a previous diagnosis of elevated lipid profiles, current use of statins or lipid-lowering medications, TG ≥ 150 mg/dl, TC ≥ 200 mg/dl, HDL-C < 50 mg/dl or LDL-C ≥ 130 mg/dl according to *the* NCEP-ATP (III) guidelines. TG: triglycerides; TC: total cholesterol; HDL-C: high-density lipoprotein cholesterol; LDL-C: low-density lipoprotein cholesterol

^†^Amino acid intake was adjusted for energy intake using the residual method

Model 1 was adjusted for age (continuous, years), years of stay in Korea (‘≤4 years, 5–9 years, ≥ 10 years), education (elementary and high school, college education and above), employment (no, yes), ever smoke (no, yes), current alcohol use (no, yes) and energy intake (continuous, kcal/d).

Model 2 was adjusted for vigorous physical activity (no, yes) in addition to covariates in model 1

Model 3 was adjusted for history of diabetes (no, yes) or hypertension (no, yes) in addition to covariates in model 2

Model 4 was adjusted for BMI (continuous, kg/m^2^) in addition to covariates in model 3

Model 5 was adjusted for minimum dietary diversity for women scores (continuous, points) in addition to covariates in model 4

Also, trends of total TG, TC, HDL-C or LDL-C were not significant with increasing tertiles of energy-adjusted tBCAA intakes (Table 3) after adjusting for age, years of stay in Korea, education, employment, ever smoked, current alcohol use, total energy intake, vigorous physical activity, history of diabetes or hypertension, BMI and MDD-W scores. For example, LS means and 95% CIs of TG by tertile distribution of energy-adjusted *t*BCAA were 89.9 (82.4, 97.4), 88.8 (81.3, 96.2) and 85.8 (78.2, 93.4) mg/dl (*P trend* = 0.45) for first, second and third tertile respectively. Similarly, LS means and 95% CIs of HDL-C by tertiles of energy-adjusted *t*BCAA intakes were 57.5 (55.3, 59.8), 59.6 (57.4, 61.8) and 57.1 (54.9, 59.4) mg/dl (*P trend* = 0.75) for first, second and third tertile respectively. Likewise, LS means and 95% CIs of total TG, TC, HDL-C, and LDL-C did not show significant trends across increasing tertiles of energy-adjusted dietary isoleucine (Table S5), leucine (Table S6) and valine (Table S7) intakes.

**Table 3:** Least-square means and 95% confidence intervals of lipid profiles by tertile distribution of energy-adjusted total BCAA intakes*

	Tertile 1	Tertile 2	Tertile 3	*P for trend*
**TG** *(mg/dl)*				
Age-adjusted	87.9 (80.1, 95.8)	89.6 (81.7, 97.5)	87.8 (79.8, 95.8)	0.98
Model 1	88.2 (80.4, 96.0)	89.4 (81.4, 97.3)	87.8 (79.9, 95.8)	0.94
Model 2	88.5 (80.7, 96.4)	89.1 (81.2, 97.0)	87.7 (79.8, 95.7)	0.88
Model 3	88.3 (80.5, 96.1)	89.2 (81.3, 97.1)	87.9 (79.9, 95.8)	0.93
Model 4	87.9 (80.4, 95.3)	89.7 (82.1, 97.2)	87.0 (79.4, 94.6)	0.87
Model 5	89.9 (82.4, 97.4)	88.8 (81.3, 96.2)	85.8 (78.2, 93.4)	0.45
**TC** *(mg/dl)*				
Age-adjusted	178.4 (172.9, 184.0)	183.5 (178.0, 189.1)	177.0 (171.3, 182.6)	0.70
Model 1	178.3 (172.8, 183.8)	184.1 (178.6, 189.6)	176.6 (171.0, 182.1)	0.64
Model 2	178.6 (173.1, 184.0)	183.9 (178.4, 189.4)	176.5 (170.9, 182.0)	0.57
Model 3	178.6 (173.1, 184.1)	183.8 (178.2, 189.3)	176.6 (171.0, 182.1)	0.59
Model 4	178.4 (173.0, 183.8)	183.9 (178.4, 189.3)	177.0 (171.4, 182.5)	0.70
Model 5	179.1 (173.6, 184.6)	183.6 (178.1, 189.0)	176.5 (171.0, 182.1)	0.48
**HDL-C** *(mg/dl)*				
Age-adjusted	57.8 (55.5, 60.1)	59.3 (57.0, 61.6)	56.9 (54.6, 59.3)	0.59
Model 1	57.7 (55.5, 60.0)	59.5 (57.3, 61.8)	56.8 (54.5, 59.1)	0.55
Model 2	57.7 (55.4, 59.9)	59.6 (57.3, 61.8)	56.8 (54.5, 59.1)	0.58
Model 3	57.7 (55.4, 60.0)	59.6 (57.3, 61.8)	56.8 (54.5, 59.1)	0.55
Model 4	57.8 (55.6, 60.0)	59.5 (57.3, 61.7)	56.9 (54.7, 59.2)	0.56
Model 5	57.5 (55.3, 59.8)	59.6 (57.4, 61.8)	57.1 (54.9, 59.4)	0.75
**LDL-C** *(mg/dl)*				
Age-adjusted	103.0 (98.1, 108.0)	106.3 (101.3, 111.3)	102.5 (97.4, 107.5)	0.86
Model 1	102.9 (98.0, 107.9)	106.7 (101.7, 111.7)	102.2 (97.2, 107.2)	0.82
Model 2	103.2 (98.3, 108.1)	106.5 (101.5, 111.4)	102.1 (97.2, 107.1)	0.75
Model 3	103.2 (98.3, 108.1)	106.4 (101.4, 111.3)	102.2 (97.2, 107.2)	0.77
Model 4	103.0 (98.2, 107.8)	106.5 (101.6, 111.3)	102.6 (97.7, 107.6)	0.91
Model 5	103.6 (98.7, 108.5)	106.2 (101.3, 111.1)	102.3 (97.2, 107.2)	0.68

*Amino acid intake was adjusted for energy intake using the residual method

TG: triglycerides; TC: total cholesterol; HDL-C: high-density lipoprotein cholesterol; LDL-C: low-density lipoprotein cholesterol

Model 1: adjusted for age (continuous, years), years of stay in Korea (≤ 4 years, 5–9 years, ≥ 10 years), education (elementary and high school, college education and above), employment (no, yes), ever smoke (no, yes), current alcohol use (no, yes) and energy intake (continuous, kcal/d)

Model 2 was adjusted for vigorous physical activity (no, yes) in addition to covariates in model 1

Model 3 was adjusted for history of diabetes (no, yes) or hypertension (no, yes) in addition to covariates in model 2

Model 4 was adjusted for BMI (continuous, kg/m^2^) in addition to covariates in model 3

Model 5 was adjusted for minimum dietary diversity for women scores (continuous, points) in addition to covariates in model 4

## Discussion

This study evaluated whether energy-adjusted dietary BCAA intakes were related to plasma lipid profiles and dyslipidaemia. There was no statistically significant relationship with individual plasma lipid profiles, but higher dietary BCAA intakes presented a statistically significant inverse trend with the prevalence of dyslipidaemia in this study. Two longitudinal studies from Japan [16, 41] have reported a direct association between higher plasma BCAA and dyslipidaemia. Similarly, increased plasma BCAA has been associated with a higher risk of hypertriglyceridemia in a sample of Germans [42] and metabolic dyslipidaemia in a Chinese population [43]. However, these report did not examine the role of dietary BCAA intakes only.

Elevated plasma BCAA profiles in metabolic disorders are well-established in the literature [6, 7, 13, 42]. However, the role of dietary BCAA intake in that association remains equivocal. Some studies have reported the relationship between dietary BCAA and metabolic disorders such as diabetes [9, 10, 44–46], obesity [8, 47–49] and insulin resistance [50, 51] with discordant conclusions. For example, in the Nurses’ Health Study and the Health Professional Follow-up study, higher dietary consumption of BCAA was associated with the risk of developing diabetes in the United States [45]. On the contrary, the Takayama study from Japan found that higher dietary BCAA was inversely related to the risk of diabetes [10]. The primary source(s) of dietary BCAA intakes differed widely between the two cohort studies. While primary sources of dietary BCAA intake were meat, milk, and fish in the report from the United States, cereals, potatoes, and starch were the primary sources of dietary BCAA intake in the Japanese study. The difference in the findings of these studies might be possible as the risk of diabetes has been reported to differ by protein sources. Animal protein consumption was associated with higher odds of diabetes, but consumption of plant protein sources was inversely related to diabetes [52, 53]. In our study, dietary BCAA were consumed primarily from both animal and vegetable food sources, including meat, grains, eggs, fish, legumes, and vegetables. However, to our knowledge, no study to date has tested whether higher dietary BCAA is associated with dyslipidaemia. Our study offers new insights into the significance of dietary BCAA in dyslipidaemia.

The statistically significant inverse trend between higher dietary BCAA intakes and the prevalence of dyslipidaemia in our study can be explained in several ways. First, the dietary environment of BCAA exposure might play a role in the inverse trend between dietary BCAA and dyslipidaemia. Some reports [8, 9, 44] have demonstrated that the dietary environment may be related to the significance of dietary BCAA intakes in the odds of metabolic disorders. For example, a report from China [9] revealed that the association between dietary BCAA and odds of diabetes might depend on the context of dietary patterns of exposure and not solely on dietary BCAA intake. In that study, significantly high odds of diabetes with higher dietary BCAA intake was observed among participants with dietary adherence to diets dense in animal sources of proteins, but not among those who adhered to plant protein sources. Similarly, another related study demonstrated the effect modification of higher meat intake on the relationship between dietary BCAA and the odds of diabetes [44]. Also, a recent meta-analysis has reported that higher dietary BCAA intakes exhibited a divergent association with odds of diabetes and obesity [54]. Protein sources of dietary BCAA in our population were from both animal and plant origins (red meat, fish, legumes and vegetables). Second, in tandem with our report, some studies have reported an inverse association between dietary BCAA supplementation and lipogenic indices in animal models [55–57]. Isoleucine supplementation was associated with lower TG accumulation in obese mice [55]. Also, leucine supplementation has been linked to reduced adiposity in high-fat diet-induced obesity among C57BL/6J male mice [56]. BCAA supplementation inhibited insulin-like growth factor receptor activation to prevent the growth of hyperlipidaemia-related colonic preneoplastic lesions in obese-hyperinsulinemic mice models [57]. Similarly, a six-week intervention trial among people with diabetes on an isocaloric protein-based diet (rich in BCAA) revealed reduced intrahepatic lipids independent of body weight through the down-regulation of lipolytic enzymes and lipogenic pathways in the adipose tissues [58]. Despite these reports, evidence alluding to the inverse association of dietary BCAA with dyslipidaemia is still evolving. Therefore, longitudinal studies and randomized controlled trials are necessary to elucidate associations of dietary BCAA intake with plasma lipid profiles and dyslipidaemia.

There are strengths and limitations worth mentioning in this study. Our report is the first (to our knowledge) to discuss the relationship between dietary BCAA and dyslipidaemia, considering diet quality. Multivariate adjustment for potential confounding minimized bias(es) in our findings, although we cannot rule out the possibility of residual or unknown confounding factors. The causal inference of our findings is limited, given that it was a cross-sectional study. A lack of statistical power to detect a significant difference for the tested associations is probable due to the study’s small size. Because the recruitment of respondents was by convenience sampling, the generalizability to the entire Filipino population may be limited. Dietary information was acquired using a single 24-hour dietary recall; therefore, the assessment of habitual dietary exposure might not be optimal. Future longitudinal studies are necessary for clarifying these associations.

## Conclusion

In this study, even though higher dietary intakes of BCAA were not associated with plasma lipid profiles, we observed a statistically significant inverse trend with the prevalence of dyslipidaemia in this sample of Filipino women in Korea.

## Electronic supplementary material

Below is the link to the electronic supplementary material.

Supplementary Material 1

## Data Availability

The data for this study cannot be made publicly available because the FiLWHEL study is still ongoing, and during the signing of consent, the respondents were not informed that their information would be stored in a publicly accessible database. However, other researchers can collaborate with the study team under approval procedures posted on the study website (www.filwhel.org). Requests to access the data may be sent to the data access committee (nutepid@gmail.com).

## Abbreviations

BCAA: Branched-chain amino acids
BMI: Body mass index
CI: Confidence interval
FiLWHEL: Filipino Women’s Diet and Health Study
HDL-C: High-density lipoprotein-cholesterol
LDL-C: Low-density lipoprotein-cholesterol
LS: Least-square
MDD-W: Minimum dietary diversity for women
NCEP-ATP: National Cholesterol Education Program Adult Treatment Panel
PR: Prevalence ratio
SD: Standard deviation
*t*bCAA: Total branched-chain amino acids
TC: Total cholesterol
TG: Triglycerides
WC: Waist circumference
