# Supplementary material for: Dietary intakes of branched-chain amino acids and plasma lipid profiles among filipino women in Korea: the Filipino Women’s Diet and Health Study (FiLWHEL)
Source: Nutr J. 2023 Jul 11;22:34. doi: 10.1186/s12937-023-00861-w (PMC10334570; doi:10.1186/s12937-023-00861-w)
Supplement: Supplementary file 1 — Supplementary Material 1 [file 12937_2023_861_MOESM1_ESM.docx]

| Table S1: Characteristics of Filipino women by tertile distribution of *isoleucine* in the FiLWHEL study | | | |
| --- | --- | --- | --- |
|  | Tertile distribution of energy-adjusted *isoleucine* intake | | |
|  | Tertile 1 | Tertile 2 | Tertile 3 |
| *N* | 141 | 141 | 141 |
| Age (years) | 34.6±8.3 | 35.5±8.1 | 35.5±7.6 |
| <35 years | 73 (51.8) | 68 (48.2) | 67 (47.5) |
| ≥35 years | 68 (48.2) | 73 (51.8) | 74 (52.5) |
| Length of stay (years) | 7.9±5.1 | 7.6±4.6 | 7.5±4.8 |
| ≤4 years | 43 (31.9) | 41 (29.9) | 40 (29.9) |
| 5 – 9 years | 43 (31.8) | 54 (39.4) | 56 (41.8) |
| ≥10 years | 49 (36.3) | 42 (30.7) | 38 (28.3) |
| Education |  |  |  |
| Elementary and high school | 45 (32.1) | 40 (28.6) | 45 (31.2) |
| College education and above | 95 (67.9) | 100 (71.4) | 95 (67.8) |
| Employment status (*Yes*) | 69 (49.3) | 78 (55.7) | 81 (57.9) |
|  |  |  |  |
| Ever smoked (*Yes*) | 12 (8.6) | 09 (6.5) | 15 (10.8) |
| Current alcohol use (*Yes*) | 79 (60.0) | 77 (56.6) | 76 (55.1) |
| Vigorous physical activity (*Yes*) | 29 (21.0) | 23 (16.8) | 27 (19.4) |
| History of diabetes* (*Yes*) | 02 (1.4) | 03 (2.1) | 05 (3.6) |
| History of hypertension* (*Yes*) | 11 (7.9) | 09 (6.4) | 09 (6.4) |
|  |  |  |  |
| BMI (kg/m^2^) | 23.7±3.7 | 23.6±3.9 | 23.5±4.1 |
| ≥ 25 kg/m^2^ | 47 (33.3) | 44 (31.2) | 37 (26.6) |
| Waist circumference (cm) | 79.4±8.8 | 79.2±9.2 | 79.4±10.1 |
| ≥ 80 cm | 61 (43.3) | 63 (44.7) | 54 (38.3) |
|  |  |  |  |
| Total energy (*kcal/d*) | 1864.7±790.9 | 1529.7±589.2 | 1818.2±601.3 |
| Dietary isoleucine intake (*g/d*)^†^ | 1.1±0.5 | 2.1±0.2 | 3.2±0.8 |
| MDD-W score | 6.0 ± 1.9 | 6.4 ± 1.7 | 6.9 ± 1.5 |
|  |  |  |  |
| TG (mg/dl) | 88.6±44.3 | 90.0±48.1 | 86.8±49.9 |
| ≥ 150mg/dl | 14 (10.0) | 14 (10.2) | 13 (9.6) |
| TC (mg/dl) | 178.3±33.5 | 183.8±34.7 | 176.9±35.2 |
| ≥ 200mg/dl | 35 (25.0) | 43 (31.4) | 33 (24.4) |
| HDL-C (mg/dl) | 57.8±13.7 | 58.5±13.2 | 57.7±14.3 |
| ≤ 50mg/dl | 40 (28.6) | 33 (24.1) | 40 (29.6) |
| LDL-C (mg/dl) | 102.8±31.3 | 107.3±29.5 | 101.8±30.5 |
| ≥ 130mg/dl | 23 (16.4) | 30 (21.9) | 23 (17.0) |
|  |  |  |  |
| Dyslipidaemia^‡^ (*Yes*) | 74 (52.5) | 69 (48.9) | 65 (46.1) |
| mean±SD for continuous variables and n (%) for categorical variables  *Self-reported clinical diagnosis or current use of medication; BMI: body mass index; MDD-W: minimum dietary diversity for women; TG: triglycerides; TC: total cholesterol; HDL-C: high-density lipoprotein cholesterol; LDL-C: low-density lipoprotein cholesterol  ^†^Amino acid intake was adjusted for energy intake using the residual method  ^‡^D*yslipidemia* was defined as one of the following conditions; a previous diagnosis of elevated lipid profiles, current use of statins or lipid-lowering medications, TG ≥ 150mg/dl, TC ≥ 200mg/dl, HDL-C <50mg/dl or LDL-C ≥ 130mg/dl according to *the* NCEP-ATP (III) guidelines. | | | |

| Table S2: Characteristics of Filipino women by tertile distribution of *leucine* intake in the FiLWHEL study | | | |
| --- | --- | --- | --- |
|  | Tertile distribution of energy-adjusted *leucine* intake | | |
|  | Tertile 1 | Tertile 2 | Tertile 3 |
| *N* | 141 | 141 | 141 |
| Age (years) | 34.6±8.3 | 35.5±8.1 | 35.5±7.6 |
| <35 years | 74 (52.5) | 70 (49.6) | 64 (45.4) |
| ≥35 years | 67 (47.5) | 71 (50.4) | 77 (54.6) |
| Length of stay (years) | 7.8±5.1 | 7.4±4.5 | 7.8±4.9 |
| ≤4 years | 45 (33.3) | 42 (30.9) | 37 (27.4) |
| 5 – 9 years | 42 (31.1) | 55 (40.4) | 56 (41.5) |
| ≥10 years | 48 (35.6) | 39 (28.7) | 42 (31.1) |
| Education |  |  |  |
| Elementary and high school | 46 (32.9) | 42 (30.0) | 42 (30.0) |
| College education and above | 94 (67.1) | 98 (70.0) | 98 (70.0) |
| Employment status (*Yes*) | 72 (51.4) | 73 (52.1) | 83 (59.3) |
|  |  |  |  |
| Ever smoked (*Yes*) | 12 (8.6) | 11 (7.9) | 13 (9.4) |
| Current alcohol use (*Yes*) | 77 (57.5) | 79 (58.1) | 76 (55.1) |
| Vigorous physical activity (*Yes*) | 30 (21.7) | 22 (16.1) | 27 (19.4) |
| History of diabetes* (*Yes*) | 02 (1.4) | 02 (1.4) | 06 (4.3) |
| History of hypertension* (*Yes*) | 11 (7.9) | 08 (5.7) | 10 (7.1) |
|  |  |  |  |
| BMI (kg/m^2^) | 23.8±3.8 | 23.3±3.5 | 23.7±4.3 |
| ≥ 25 kg/m^2^ | 50 (35.5) | 39 (27.7) | 39 (28.1) |
| Waist circumference (cm) | 79.7±8.7 | 78.6±8.6 | 79.7±10.6 |
| ≥ 80 cm | 65 (46.1) | 57 (40.4) | 56 (39.7) |
|  |  |  |  |
| Total energy (*kcal/d*) | 1885.8±786.8 | 1499.0±568.7 | 1827.7±608.6 |
| Dietary leucine intake (*g/d*)^†^ | 1.9±0.8 | 3.6±0.4 | 5.6±1.4 |
| MDD-W score | 6.1 ± 1.9 | 6.4 ± 1.7 | 6.8 ± 1.5 |
|  |  |  |  |
| TG (mg/dl) | 89.0±44.1 | 88.3±45.7 | 88.1±52.5 |
| ≥ 150mg/dl | 14 (10.0) | 12 (8.6) | 15 (11.3) |
| TC (mg/dl) | 178.7±34.7 | 182.0±33.9 | 178.3±35.1 |
| ≥ 200mg/dl | 35 (25.0) | 43 (30.9) | 33 (24.8) |
| HDL-C (mg/dl) | 57.7±13.5 | 59.1±14.2 | 57.3±13.4 |
| ≤ 50mg/dl | 41 (29.3) | 32 (23.0) | 40 (30.1) |
| LDL-C (mg/dl) | 103.2±31.7 | 105.3±29.5 | 103.4±30.2 |
| ≥ 130mg/dl | 23 (16.3) | 29 (20.9) | 24 (18.1) |
|  |  |  |  |
| Dyslipidaemia^‡^ (*Yes*) | 75 (53.2) | 69 (48.9) | 64 (45.4) |
| mean±SD for continuous variables and n (%) for categorical variables  *Self-reported clinical diagnosis or current use of medication; BMI: body mass index; MDD-W: minimum dietary diversity for women; TG: triglycerides; TC: total cholesterol; HDL-C: high-density lipoprotein cholesterol; LDL-C: low-density lipoprotein cholesterol  ^†^Amino acid intake was adjusted for energy intake using the residual method  ^‡^D*yslipidemia* was defined as one of the following conditions; a previous diagnosis of elevated lipid profiles, current use of statins or lipid-lowering medications, TG ≥ 150mg/dl, TC ≥ 200mg/dl, HDL-C <50mg/dl or LDL-C ≥ 130mg/dl according to *the* NCEP-ATP (III) guidelines. | | | |

| Table S3: Characteristics of Filipino women by tertile distribution of *valine* intake in the FiLWHEL study | | | |
| --- | --- | --- | --- |
|  | Tertile distribution of energy-adjusted *valine* intake | | |
|  | Tertile 1 | Tertile 2 | Tertile 3 |
| *N* | 141 | 141 | 141 |
| Age (years) | 34.2±8.2 | 36.0±8.0 | 35.4±7.6 |
| <35 years | 77 (54.6) | 64 (45.4) | 67 (47.5) |
| ≥35 years | 64 (45.4) | 77 (54.6) | 74 (52.5) |
| Length of stay (years) | 7.7±5.0 | 7.5±4.6 | 7.8±4.9 |
| ≤4 years | 44 (32.6) | 41 (30.2) | 39 (28.9) |
| 5 – 9 years | 45 (33.3) | 53 (39.0) | 55 (40.7) |
| ≥10 years | 46 (34.1) | 42 (30.8) | 41 (30.4) |
| Education |  |  |  |
| Elementary and high school | 46 (32.9) | 39 (28.1) | 45 (31.9) |
| College education and above | 94 (67.1) | 100 (71.9) | 96 (68.1) |
| Employment status (*Yes*) | 69 (48.6) | 79 (56.8) | 81 (57.5) |
|  |  |  |  |
| Ever smoked (*Yes*) | 13 (9.4) | 08 (5.8) | 15 (10.7) |
| Current alcohol use (*Yes*) | 77 (57.5) | 79 (58.1) | 76 (55.1) |
| Vigorous physical activity (*Yes*) | 31 (22.5) | 21 (15.4) | 27 (19.3) |
| History of diabetes* (*Yes*) | 02 (1.4) | 02 (1.4) | 06 (4.3) |
| History of hypertension* (*Yes*) | 10 (7.1) | 11 (7.9) | 08 (5.7) |
|  |  |  |  |
| BMI (kg/m^2^) | 23.6±3.7 | 23.6±3.7 | 23.6±4.2 |
| ≥ 25 kg/m^2^ | 45 (31.9) | 44 (31.2) | 39 (28.1) |
| Waist circumference (cm) | 79.1±8.7 | 79.4±9.0 | 79.5±10.3 |
| ≥ 80 cm | 57 (40.4) | 65 (46.1) | 56 (39.7) |
|  |  |  |  |
| Total energy (*kcal/d*) | 1841.2±801.3 | 1553.0±571.9 | 1818.3±616.2 |
| Dietary valine intake (*g/d*)^†^ | 1.3±0.6 | 2.4±0.2 | 3.7±0.9 |
| MDD-W score | 6.0 ± 1.9 | 6.4 ± 1.7 | 6.9 ± 1.5 |
|  |  |  |  |
| TG (mg/dl) |  |  |  |
| ≥ 150mg/dl | 86.9±44.1 | 90.3±46.4 | 88.2±51.7 |
| TC (mg/dl) | 13 (9.3) | 14 (10.1) | 14 (10.5) |
| ≥ 200mg/dl | 178.1±34.1 | 182.1±33.7 | 178.8±35.8 |
| HDL-C (mg/dl) | 36 (25.7) | 41 (29.7) | 34 (25.4) |
| ≤ 50mg/dl | 57.9±13.8 | 58.9±13.5 | 57.2±13.8 |
| LDL-C (mg/dl) | 41 (29.3) | 33 (23.9) | 39 (29.1) |
| ≥ 130mg/dl | 102.8±31.9 | 105.1±28.9 | 104.0±30.7 |
|  | 24 (17.0) | 28 (20.3) | 24 (17.9) |
|  |  |  |  |
| Dyslipidaemia^‡^ (*Yes*) | 76 (53.9) | 67 (47.5) | 65 (46.1) |
| mean±SD for continuous variables and n (%) for categorical variables  *Self-reported clinical diagnosis or current use of medication; BMI: body mass index; MDD-W: minimum dietary diversity for women; TG: triglycerides; TC: total cholesterol; HDL-C: high-density lipoprotein cholesterol; LDL-C: low-density lipoprotein cholesterol  ^†^Amino acid intake was adjusted for energy intake using the residual method  ^‡^D*yslipidemia* was defined as one of the following conditions; a previous diagnosis of elevated lipid profiles, current use of statins or lipid-lowering medications, TG ≥ 150mg/dl, TC ≥ 200mg/dl, HDL-C <50mg/dl or LDL-C ≥ 130mg/dl according to *the* NCEP-ATP (III) guidelines. | | | |

| Table S4: Percentage contribution (%) of foods groups to dietary BCAA intakes in the FiLWHEL study | | | |
| --- | --- | --- | --- |
| Food Groups | Isoleucine | Leucine | Valine |
| Red Meat | 23.8 | 24.8 | 24.8 |
| Grains | 19.2 | 20.8 | 17.9 |
| Fish | 11.7 | 10.8 | 11.1 |
| Eggs | 9.6 | 8.7 | 10.0 |
| Poultry meat | 8.8 | 8.5 | 8.0 |
| Sea foods | 5.5 | 5.7 | 5.2 |
| Legumes | 4.9 | 4.8 | 4.9 |
| Vegetables | 4.1 | 3.5 | 4.8 |

| Table S5: Least-square means and 95% confidence intervals of lipid profiles by tertile distribution of energy-adjusted isoleucine intakes* | | | | |
| --- | --- | --- | --- | --- |
|  | Tertile 1 | Tertile 2 | Tertile 3 | *P trend* |
| TG *(mg/dl)* |  |  |  |  |
| Age-adjusted | 89.1 (81.3, 96.9) | 89.6 (81.7, 97.5) | 86.7 (78.7, 94.6) | 0.67 |
| Model 1 | 89.3 (81.5, 97.2) | 89.3 (81.3, 97.3) | 86.7 (78.7, 94.6) | 0.64 |
| Model 2 | 89.5 (81.7, 97.4) | 89.1 (81.2, 97.1) | 86.7 (78.7, 94.6) | 0.61 |
| Model 3 | 89.4 (81.5, 97.2) | 89.2 (81.2, 97.1) | 86.8 (78.9, 94.7) | 0.65 |
| Model 4 | 89.1 (81.7, 96.6) | 88.6 (81.0, 96.1) | 86.8 (79.2, 94.4) | 0.66 |
| Model 5 | 91.2 (83.7, 98.6) | 87.7 (80.2, 95.2) | 85.6 (78.0, 93.1) | 0.31 |
|  |  |  |  |  |
| TC *(mg/dl)* |  |  |  |  |
| Age-adjusted | 178.9 (173.4, 184.5) | 183.3 (177.6, 188.9) | 176.8 (171.2, 182.4) | 0.60 |
| Model 1 | 178.6 (173.1, 184.1) | 184.0 (178.4, 189.6) | 176.4 (170.9, 182.0) | 0.58 |
| Model 2 | 178.7 (173.3. 184.2) | 183.8 (178.3, 189.4) | 176.4 (170.9, 181.9) | 0.55 |
| Model 3 | 178.8 (173.3, 184.2) | 183.8 (178.2, 189.4) | 176.4 (170.9, 182.0) | 0.56 |
| Model 4 | 178.6 (173.2, 184.0) | 183.5 (178.0, 189.0) | 177.1 (171.6, 182.7) | 0.70 |
| Model 5 | 179.4 (173.9, 184.8) | 183.2 (177.7, 188.7) | 176.7 (171.2, 182.3) | 0.49 |
|  |  |  |  |  |
| HDL-C *(mg/dl)* |  |  |  |  |
| Age-adjusted | 57.9 (55.7, 60.2) | 58.4 (56.1, 60.7) | 57.7 (55.4, 60.0) | 0.90 |
| Model 1 | 57.9 (55.6, 60.1) | 58.5 (56.2, 60.8) | 57.7 (55.4, 60.0) | 0.90 |
| Model 2 | 57.8 (55.6, 60.1) | 58.6 (56.2, 60.9) | 57.7 (55.4, 60.0) | 0.92 |
| Model 3 | 57.9 (55.6, 60.1) | 58.5 (56.2, 60.8) | 57.6 (55.3, 59.9) | 0.88 |
| Model 4 | 57.9 (55.7, 60.1) | 58.7 (56.5, 60.9) | 57.6 (55.4, 59.9) | 0.84 |
| Model 5 | 57.7 (55.4, 60.0) | 58.8 (56.6, 61.1) | 57.8 (55.5, 60.0) | 0.95 |
|  |  |  |  |  |
| LDL-C *(mg/dl)* |  |  |  |  |
| Age-adjusted | 103.2 (98.2, 108.2) | 106.9 (101.9, 111.9) | 101.7 (96.7, 106.8) | 0.69 |
| Model 1 | 102.8 (97.9, 107.8) | 107.6 (102.6, 112.6) | 101.4 (96.4, 106.4) | 0.69 |
| Model 2 | 103.0 (98.1, 107.9) | 107.5 (102.5, 112.4) | 101.4 (96.4, 106.4) | 0.65 |
| Model 3 | 103.0 (98.1, 107.9) | 107.4 (102.4, 112.4) | 101.4 (96.5, 106.4) | 0.66 |
| Model 4 | 102.9 (98.1, 107.7) | 107.1 (102.2, 112.0) | 102.2 (97.2, 107.1) | 0.84 |
| Model 5 | 103.5 (98.6, 108.3) | 106.8 (101.9, 111.7) | 101.8 (96.9, 106.7) | 0.63 |
| *Amino acid intake was adjusted for energy intake using the residual method  TG: triglycerides; TC: total cholesterol; HDL-C: high-density lipoprotein cholesterol; LDL-C: low-density lipoprotein cholesterol  Model 1: adjusted for age (continuous, years), years of stay in Korea (≤4 years, 5-9 years, ≥10 years), education (elementary and high school, college education and above), employment (no, yes), ever smoke (no, yes), current alcohol use (no, yes) and energy intake (continuous, kcal/d).  Model 2 was adjusted for vigorous physical activity (no, yes) in addition to covariates in model 1  Model 3 was adjusted for history of diabetes (no, yes) or hypertension (no, yes) in addition to covariates in model 2.  Model 4 was adjusted for BMI (continuous, kg/m^2^) in addition to covariates in model 3.  Model 5 was adjusted for minimum dietary diversity for women scores (continuous, points) in addition to covariates in model 4. | | | | |

| Table S6: Least-square means and 95% confidence intervals of lipid profiles by tertile distribution of energy-adjusted *leucine* intakes* | | | | |
| --- | --- | --- | --- | --- |
|  | Tertile 1 | Tertile 2 | Tertile 3 | *P trend* |
| TG *(mg/dl)* |  |  |  |  |
| Age-adjusted | 89.6 (81.8, 97.4) | 87.8 (80.0, 95.7) | 87.9 (79.9, 95.9) | 0.77 |
| Model 1 | 90.3 (82.5, 98.2) | 86.8 (78.8, 94.8) | 88.3 (80.3, 96.3) | 0.73 |
| Model 2 | 90.6 (82.8, 98.4) | 86.5 (78.5, 94.5) | 88.3 (80.3, 96.3) | 0.70 |
| Model 3 | 90.4 (82.6, 98.2) | 86.7 (78.7, 94.7) | 88.3 (80.3, 96.2) | 0.71 |
| Model 4 | 89.8 (82.3, 97.2) | 87.6 (80.0, 95.2) | 87.2 (79.5, 94.8) | 0.63 |
| Model 5 | 91.8 (84.3, 99.3) | 86.7 (79.1, 94.2) | 86.0 (78.4, 93.6) | 0.30 |
|  |  |  |  |  |
| Total cholesterol *(mg/dl)* |  |  |  |  |
| Age-adjusted | 179.5 (173.9, 185.0) | 181.4 (175.8, 187.0) | 178.1 (172.4, 183.8) | 0.73 |
| Model 1 | 179.3 (173.8, 184.8) | 181.6 (176.0, 187.2) | 178.0 (172.4, 183.6) | 0.73 |
| Model 2 | 179.6 (174.1, 185.0) | 181.4 (175.8, 187.0) | 178.0 (172.4, 183.6) | 0.68 |
| Model 3 | 179.6 (174.1, 185.1) | 181.2 (175.6, 186.8) | 178.2 (172.6, 183.8) | 0.72 |
| Model 4 | 179.3 (173.9, 184.7) | 181.5 (175.9, 187.0) | 178.5 (172.9, 184.1) | 0.83 |
| Model 5 | 180.0 (174.5, 185.5) | 181.1 (175.6, 186.7) | 178.1 (172.5, 183.7) | 0.61 |
|  |  |  |  |  |
| HDL-C *(mg/dl)* |  |  |  |  |
| Age-adjusted | 57.8 (55.5, 60.1) | 59.0 (56.7, 61.2) | 57.3 (54.9, 59.6) | 0.74 |
| Model 1 | 57.6 (55.4, 59.9) | 59.3 (57.0, 61.6) | 57.1 (54.7, 59.4) | 0.70 |
| Model 2 | 57.6 (55.3, 59.9) | 59.4 (57.1, 61.7) | 57.1 (54.7, 59.4) | 0.72 |
| Model 3 | 57.6 (55.4, 59.9) | 59.3 (57.0, 61.6) | 57.0 (54.7, 59.3) | 0.71 |
| Model 4 | 57.8 (55.6, 60.0) | 59.1 (56.9, 61.4) | 57.3 (55.0, 59.6) | 0.74 |
| Model 5 | 57.5 (55.3, 59.8) | 59.3 (57.0, 61.5) | 57.5 (55.2, 59.7) | 0.93 |
|  |  |  |  |  |
| LDL-C *(mg/dl)* |  |  |  |  |
| Age-adjusted | 103.7 (98.8, 108.7) | 104.9 (99.9, 109.9) | 103.3 (98.2, 108.4) | 0.89 |
| Model 1 | 103.6 (98.7, 108.6) | 104.9 (99.9, 110.0) | 103.3 (98.3, 108.3) | 0.92 |
| Model 2 | 103.8 (98.9, 108.8) | 104.7 (99.7, 109.7) | 103.3 (98.3, 108.3) | 0.87 |
| Model 3 | 103.9 (98.9, 108.8) | 104.5 (99.5, 109.6) | 103.5 (98.4, 108.5) | 0.91 |
| Model 4 | 103.5 (98.7, 108.4) | 104.8 (99.9, 109.7) | 103.8 (98.8, 108.7) | 0.95 |
| Model 5 | 104.2 (99.2, 109.1) | 104.5 (99.6, 109.5) | 103.4 (98.5, 108.4) | 0.83 |
| *Amino acid intake was adjusted for energy intake using the residual method  TG: triglycerides; TC: total cholesterol; HDL-C: high-density lipoprotein cholesterol; LDL-C: low-density lipoprotein cholesterol  Model 1: adjusted for age (continuous, years), years of stay in Korea (≤4 years, 5-9 years, ≥10 years), education (elementary and high school, college education and above), employment (no, yes), ever smoke (no, yes), current alcohol use (no, yes) and energy intake (continuous, kcal/d).  Model 2 was adjusted for vigorous physical activity (no, yes) in addition to covariates in model 1  Model 3 was adjusted for history of diabetes (no, yes) or hypertension (no, yes) in addition to covariates in model 2.  Model 4 was adjusted for BMI (continuous, kg/m^2^) in addition to covariates in model 3.  Model 5 was adjusted for minimum dietary diversity for women scores (continuous, points) in addition to covariates in model 4. | | | | |

| Table S7: Least-square means and 95% confidence intervals of lipid profiles by tertile distribution of energy-adjusted valine intakes* | | | | |
| --- | --- | --- | --- | --- |
|  | Tertile 1 | Tertile 2 | Tertile 3 | *P trend* |
| TG *(mg/dl)* |  |  |  |  |
| Age-adjusted | 87.7 (79.9, 95.5) | 89.5 (81.6, 97.4) | 88.2 (80.2, 96.2) | 0.93 |
| Model 1 | 87.8 (79.9, 95.6) | 89.4 (81.5, 97.4) | 88.2 (80.2, 96.2) | 0.95 |
| Model 2 | 88.1 (80.3, 95.9) | 89.1 (81.2, 97.1) | 88.2 (80.2, 96.1) | 0.99 |
| Model 3 | 88.0 (80.2, 95.8) | 89.0 (81.0, 97.0) | 88.4 (80.4, 96.4) | 0.95 |
| Model 4 | 88.1 (80.7, 95.5) | 88.6 (81.0, 96.2) | 87.9 (80.3, 95.5) | 0.99 |
| Model 5 | 90.1 (82.6, 97.6) | 88.1 (81.1, 95.6) | 86.3 (78.7, 93.9) | 0.49 |
|  |  |  |  |  |
| Cholesterol *(mg/dl)* |  |  |  |  |
| Age-adjusted | 179.2 (173.6, 184.7) | 181.0 (175.4, 186.6) | 178.9 (173.2, 184.5) | 0.93 |
| Model 1 | 178.9 (173.4, 184.4) | 181.4 (175.8, 187.0) | 178.7 (173.1, 184.3) | 0.94 |
| Model 2 | 179.2 (173.7. 184.7) | 181.1 (175.5, 186.7) | 178.7 (173.1, 184.2) | 0.88 |
| Model 3 | 179.2 (173.7, 184.7) | 181.1 (175.5, 186.6) | 178.7 (173.1, 184.3) | 0.90 |
| Model 4 | 179.2 (173.8, 184.6) | 180.8 (175.3, 186.3) | 179.3 (173.7, 184.9) | 0.99 |
| Model 5 | 180.0 (174.5, 185.5) | 180.6 (175.1 186.1) | 178.7 (173.1, 184.3) | 0.75 |
|  |  |  |  |  |
| HDL-C *(mg/dl)* |  |  |  |  |
| Age-adjusted | 58.0 (55.8, 60.3) | 58.8 (56.5, 61.1) | 57.2 (54.9, 59.6) | 0.62 |
| Model 1 | 58.0 (55.7, 60.3) | 58.8 (56.5, 61.1) | 57.2 (54.9, 59.5) | 0.60 |
| Model 2 | 58.0 (55.7, 60.2) | 58.9 (56.6, 61.2) | 57.2 (54.9, 59.5) | 0.62 |
| Model 3 | 58.0 (55.7, 60.3) | 58.9 (56.6, 61.2) | 57.1 (54.8, 59.4) | 0.59 |
| Model 4 | 58.0 (55.8, 60.2) | 59.0 (56.8, 61.3) | 57.2 (55.0, 59.5) | 0.63 |
| Model 5 | 57.7 (55.5, 59.9) | 59.1 (56.9, 61.3) | 57.4 (55.2, 59.7) | 0.84 |
|  |  |  |  |  |
| LDL-C *(mg/dl)* |  |  |  |  |
| Age-adjusted | 103.6 (98.6, 108.6) | 104.3 (99.3, 109.3) | 104.0 (98.9, 109.1) | 0.92 |
| Model 1 | 103.4 (98.4, 108.3) | 104.6 (99.6, 109.7) | 103.9 (98.9, 108.9) | 0.89 |
| Model 2 | 103.6 (98.7, 108.6) | 104.4 (99.4, 109.4) | 103.9 (98.9, 108.8) | 0.95 |
| Model 3 | 103.6 (98.7, 108.5) | 104.3 (99.3, 109.3) | 103.9 (98.9, 108.9) | 0.93 |
| Model 4 | 103.6 (98.8, 108.4) | 104.1 (99.2, 109.0) | 104.5 (99.5, 109.4) | 0.80 |
| Model 5 | 104.2 (99.3, 109.1) | 103.9 (99.0, 108.8) | 104.0 (99.0, 109.0) | 0.95 |
| *Amino acid intake was adjusted for energy intake using the residual method  TG: triglycerides; TC: total cholesterol; HDL-C: high-density lipoprotein cholesterol; LDL-C: low-density lipoprotein cholesterol  Model 1: adjusted for age (continuous, years), years of stay in Korea (≤4 years, 5-9 years, ≥10 years), education (elementary and high school, college education and above), employment (no, yes), ever smoke (no, yes), current alcohol use (no, yes) and energy intake (continuous, kcal/d).  Model 2 was adjusted for vigorous physical activity (no, yes) in addition to covariates in model 1  Model 3 was adjusted for history of diabetes (no, yes) or hypertension (no, yes) in addition to covariates in model 2.  Model 4 was adjusted for BMI (continuous, kg/m^2^) in addition to covariates in model 3.  Model 5 was adjusted for minimum dietary diversity for women scores (continuous, points) in addition to covariates in model 4. | | | | |
